# Evaluation of the Nematicidal and Phytotoxic Potential of *Ricinus communis*, *Cosmos bipinnatus*, and *Tagetes erecta* Plant Extracts in Tomato

**DOI:** 10.3390/plants15121872

**Published:** 2026-06-17

**Authors:** Jeisel Delgado Flores, María E. Jaramillo Flores, Raúl J. Delgado Macuil

**Affiliations:** 1 Instituto Politécnico Nacional, Centro de Investigación en Biotecnología Aplicada, Carretera Est. Santa Inés Tecuexcomac-Tepetitla, Km 1.5 Tepetitla de Lardizabal, Tlaxcala 90700, Mexico; jdelgadof1700@alumno.ipn.mx; 2 Instituto Politécnico Nacional, Escuela Nacional de Ciencias Biológicas, Ingeniería Bioquímica, Ciudad de México 07738, Mexico; mjarfl@ipn.mx

**Keywords:** plant extracts, root-knot nematode, phytoparasites control, tomato seed germination

## Abstract

Plant-parasitic nematodes cause great damage to tomato production, which consequently has a deleterious economic impact on producers. For decades, parasitic control has relied on chemical nematicides with toxic environmental effects. Currently, an emerging option is the use of plant extracts, which offer suitable control of these plant pathogens without causing environmental damage. Therefore, in this study we examine the activity of three plant extracts to reduce the population of plant-parasitic nematodes that cause root gall formation in tomato plants, using in vitro tests and greenhouse experiments. First, a molecular identification of the nematodes was performed, identifying the pathogen species as *Meloidogyne incognita* and *Nacobbus aberrans*. Then, different extract concentrations were obtained from plant tissues of *Cosmos bipinnatus*, *Ricinus communis*, and *Tagetes erecta*. It was found that *C. bipinnatus* extract presented the highest nematode immobility, followed by *T. erecta*, reducing populations by up to 95 to 100%. Nevertheless, according to the germination tests on tomato seeds, the extracts also seem to have a minimal phytotoxic effect on the development/first growth stage of tomato plants under greenhouse conditions.

## 1. Introduction

Plant-parasitic nematodes (PPN) of the genus *Meloidogyne* spp. and *Nacobbus aberrans* can spread on plants by forming galls on their roots, mainly affecting crops of tomato (*Solanum lycopersicum*), potato (*Solanum tuberosum*), bean (*Phaseolus vulgaris*), and chili (*Capsicum annuum*), among others [1]. Tomato is the most consumed vegetable in the world, being cultivated throughout the year; however, it is one of the most affected by PPN, causing annual losses between 80 and 110 billion dollars worldwide [2]. The severity of crop damage and yield reduction is influenced by nematode species, but also involves variables such as host resistance, crop rotation, time of year, and soil type [3]. In addition, *Meloidogyne* spp. and *N. aberrans* can interact with other soil-borne pathogens, thereby increasing host plant damage and crop losses [4]. Globally, these nematodes cause 10–12% of tomato crop damage and loss of its 243 million ton production [5]. Both nematode species are distributed throughout the Americas, including Mexico, where the states affected by these parasites are Sinaloa, San Luis Potosí, Michoacán, Zacatecas, Jalisco, Puebla, and Tlaxcala [5]. Regarding the control methods, there are chemical nematicides derived from methyl bromide, chloropicrin, and methyl isocyanate employed as volatile fumigant compounds, as well as non-volatile ones such as organophosphates (ethoprophos and phenammphos) and carbamates (aldicarb, carbofuran and oxamyl) [6]. The Environmental Protection Agency (EPA) has restricted the use of many of these products due to their environmental toxicity [7,8], from which the uncontrolled use of chemical nematicides has caused a severe environmental contamination, that has extended to food, agricultural soils, and water reservoirs [9]. The presence of pesticide residues in food has caused health concerns, as they can show carcinogenic, cytotoxic, and mutagenic effects. Furthermore, nematicidal products are not specific and are often dispersed on crops in different ways (irrigation or spraying), affecting fauna and microorganisms that can be beneficial to plants [9,10]. Other management strategies include cultural practices such as rotation, mixed crops, resistant plants, biological control, and natural bioactive products [11]. Crop rotation and mixing are among the best strategies to reduce populations of PPN [12], but their practice implies crop area reduction and competition between plants [13]. Biological control comprises a wide range of formulated products, with microorganisms including bacteria, fungi, viruses, protozoa, and algae used to attack nematode populations, highlighting *Trichoderma* sp., *Bacillus megaterium*, and *Ascophyllum nodosum*. However, field evaluation has shown inconsistent efficiencies, since nematicidal effects on strains depend on the region where they are isolated, underscoring the importance of characterizing strains adapted to local conditions to enhance control efficiency [14]. The use of bioactive compounds from plant extracts represents one of the most promising strategies for managing PPN, without affecting the environment, due to their biodegradability and low toxicity. Some of the main advantages of their use in organic agriculture include no resistance effects, improved growth and yield, and null attack on non-target organisms [15,16]. For instance, the genus *Tagetes* (Asteraceae) has shown a notable nematicidal effect, in association with the rotation of tomato, potato, rose, and cucumber crops, decreasing the populations of *Meloidogyne* spp. and *N. aberrans*. This effect is attributed to the presence of secondary metabolites such as the thiophene group, produced mainly in the plant root when it detects the presence of phytoparasites, activating its production as a protection mechanism [17,18]. Aballay & Insunza (2002) reported the effect of eight antagonistic plant species against the nematode *Xiphinema index*, finding that *Cosmos bipinnatus*, added as green manure, can reduce the nematode population, this effect was attributed to secondary metabolites such as phenols and flavonoids present throughout the plant, especially in the flowers, characterized by their purple color [19]. Likewise, *Ricinus communis* is also well-known by the toxic effect of its seeds, caused by the presence of highly toxic plant albumins such as ricin and ricinin. Some authors indicate that these components can be beneficial in combating fungal, bacterial, and viral diseases, as well as insect pests and phytoparasites affecting various commercially important crops [20,21]. Thus, this work aimed to determine the bionematicidal effect of complete extracts of *Ricinus communis*, *Cosmos bipinnatus,* and *Tagetes erecta* against two species of PPN, molecularly identified as *Meloidogyne incognita* and *Nacobbus aberrans*. These extracts were obtained from different plant tissues, using three organic solvents, establishing the minimum inhibitory concentration to reduce the nematode population causing gall formation in tomato plants.

## 2. Results

### 2.1. Nematode Molecular Identification

**Figure 1:**
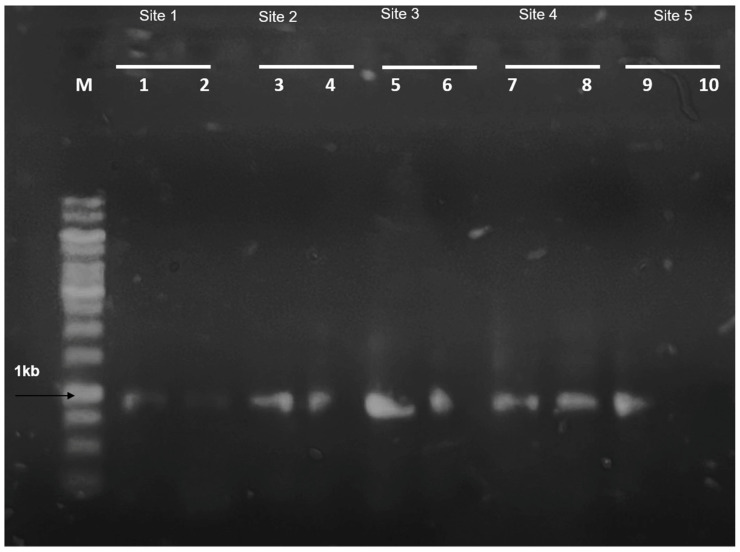
Visualization of amplified PCR products from extracted DNA from female nematodes. Lane M: molecular weight marker; lanes 1 and 2, sampling site 1; lanes 3 and 4, sampling site 2; lanes 5 and 6, sampling site 3; lanes 7 and 8, sampling site 4; lanes 9 and 10, sampling site 5.

Female nematodes were isolated from galls of tomato roots, collected from a greenhouse producing the tomato hybrid V427. Genomic DNA was extracted from 10 nematode isolates, where approximately 50 μg of high-quality DNA was obtained to perform PCRs for identification of Nematoda phylum. Agarose gel analysis of the PCR-amplified DNA product showed a 900 bp band, as observed in Figure 1 [22,23].

The PCR products were sequenced, obtaining eight read sequences used to perform Primer-BLAST analysis in the NCBI online bioinformatic tool, showing high match alignment with *Meloidogyne incognita* and *Nacobbus aberrans*, with similarities ranging from 99.5 to 100%, as shown in Table 1.

**Figure 2:**
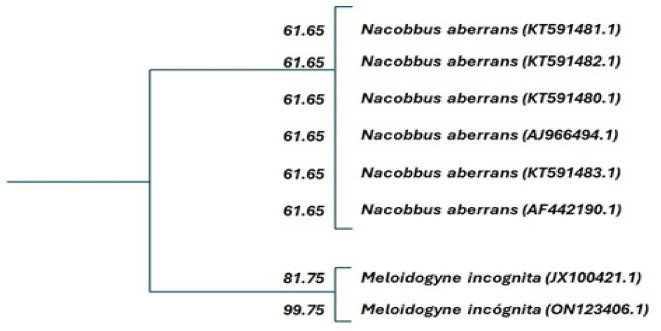
Phylogenetic tree generated from 8 nematode isolates obtained from tomato root galls.

Moreover, a phylogenetic tree was constructed from the BLAST analysis, involving eight nucleotide sequences. Gapped positions were eliminated, allowing us to establish a differentiation of the isolated nematodes. Sequences were clustered within the nematode phylum, divided in both species, as presented in the phylogenetic tree (Figure 2).

### 2.2. Molecular Identification of Collected Plants

**Figure 3:**
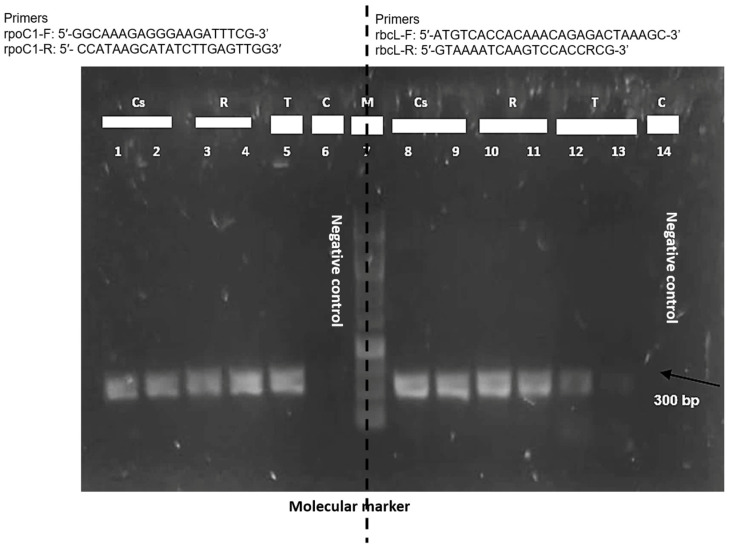
PCR amplification products of the isolated DNA from the collected plants. Five sites Cs: Mirasol, R: Higuerilla, T: Cempasuchil, C: control, and M: marker.

Three plants commonly known as Higuerilla, Cempasuchil, and Mirasol were colleted from different regions of Tlaxcala, Mexico. The three collected species showed well-defined morphological characteristics that coincided with those reported elsewhere. To authenticate these species, a molecular identification was performed from 11 plant isolates, which were divided by two primer pairs used for identification, 5 for rbcL and 6 for rpoC. The DNA extraction yielded 50 μg from plant isolates, and the corresponding PCR amplification showed a band around 300 bp [24], as observed in Figure 3.

Both primer pairs used to molecularly identify the three collected plants [24] proved to be functional, showing a 99 to 100% match for the three plant species (Table 2). In the case of the *rpoC1* primers, the PCR amplification was positive for the three species (lanes 1 to 5), whereas for the *rbcL* primer, amplification was only obtained for the Mirasol (*C. bipinnatus*) and Higuerilla (*R. communis*) plants; while for cempasuchil (*T. erecta*) (lanes 12 and 13) no marked separation of PCR products was observed. The analyzed sequences from the three plants are shown in Figure S1 (Supplementary Material).

### 2.3. In Vitro Inhibition Assays of Plant Extracts Against Nematodes

**Figure 4:**
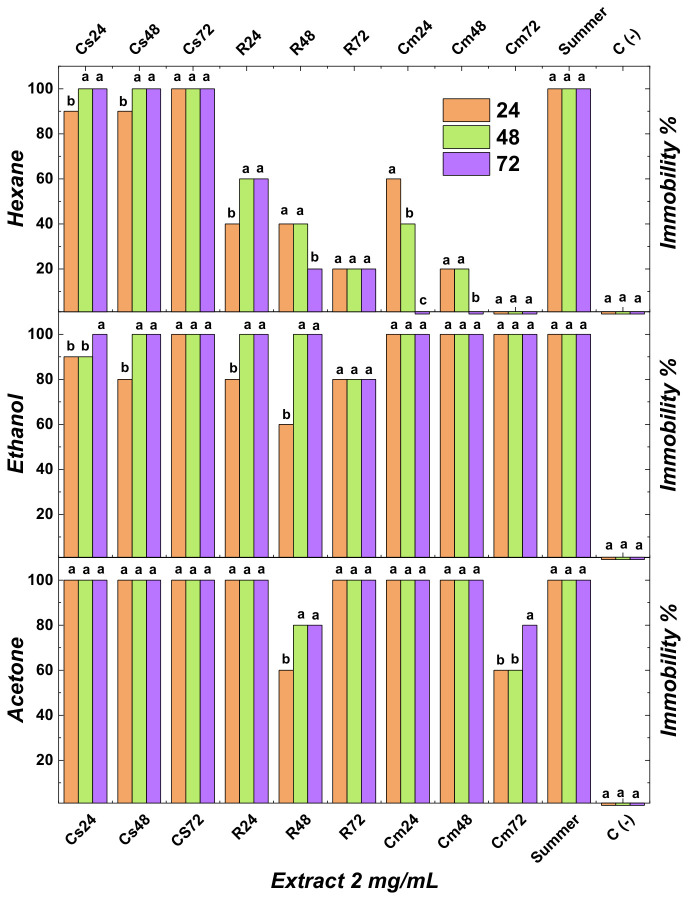
In vitro inhibition assays of plant extracts against PPN. Maximum mortality of second-stage juveniles is observed in relation to the concentration [2 mg/mL] and exposure time (24, 48 and 72 h maceration) of each evaluated extract (Cs: *C. bipinnatus*, R: *R. communis*, Cm: *T. erecta*). Data shown correspond to the average of all values. According to Tukey’s test, the same letter indicates data in each row is not significantly different (*p* ≤ 0.05), whereas different lowercase letters (a–c) for the same test indicate significant (*p* < 0.05) differences between treatments.

The bionematicidal effect of *C. bipinnatus*, *T. erecta*, and *R. communis* plant extracts was characterized as a function of nematode immobilization. A maximum immobilization effect of 100% at 24 h was obtained with the plant extracts (2 mg/mL concentration) obtained with acetone and ethanol, while those obtained with hexane showed lower immobilization yields (60%) up to 72 h (Figure 4).

**Figure 5:**
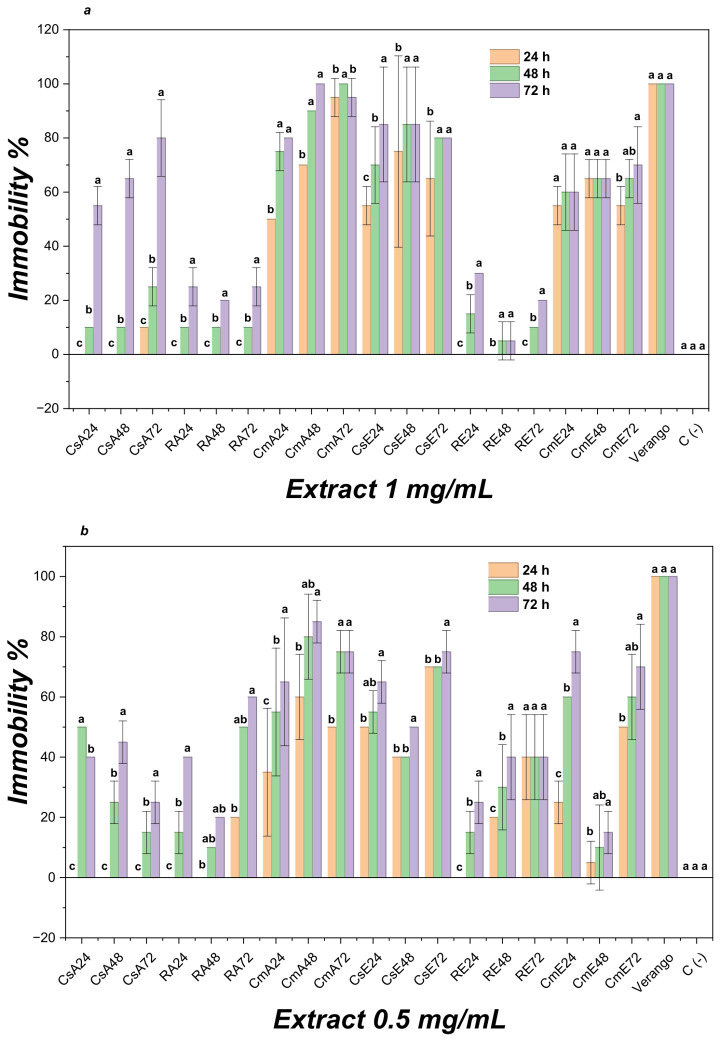
Nematicidal effect of plants extracts at (**a**) 1 mg/mL and (**b**) 0.5 mg/mL on the immobility of parasitic nematodes after different exposure times. Data shown correspond to the average of all values ± sd. According to Tukey’s test, the same letter indicates data in each row is not significantly different (*p* ≤ 0.05), whereas different lowercase letters (a–c) for the same test indicate significant (*p* < 0.05) differences between treatments. Cs: *C. bipinnatus.* R: *R. communis*, Cm: *T. erecta*. Letters A and E indicate the used solvent, included in the X-axis key (A: acetone and E: ethanol), and numbers indicate the hours of maceration with the corresponding solvent.

The extracts obtained with acetone and ethanol at 24 h showed the maximal immobility effects; thus, these were used for the next experiments, now reducing the concentrations to 1 and 0.5 mg/mL (Figure 5). As observed in Figure 5a, the maximum immobility effect was found with the *T. erecta* extract (1 mg/mL), obtained with acetone at 48 and 72 h of maceration, reaching 100% nematode immobility at 72 h of contact. Regarding *C. bipinnatus*, the highest nematicidal effect was found with ethanol extracts at 24 and 48 h maceration, showing 85% immobility at 72 h of contact, while *R. communis* decreased its effectiveness to 60–80%.

Treatments using the 0.5 mg/mL concentration of plant extracts showed the highest immobility up to 72 h, with RA48 as the only treatment that reached 85% nematode immobility, while the rest of the treatments showed immobility effects below 75%.

**Figure 6:**
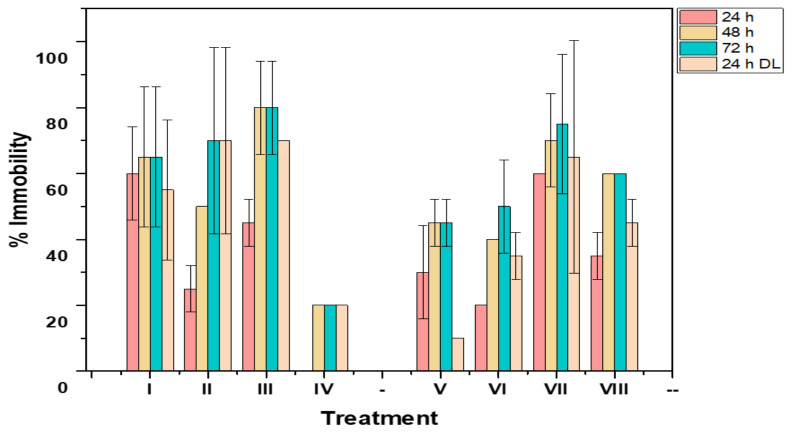
Effect of combined plant extracts on the immobility of root knot nematodes isolated from tomato plants. I: RA24 (20%) + CmA48 (10%), II: RA24 (20%) + CsA48 (20%), III: CmA 48 (10%) + CsA48 (20%), IV: CmA48 (10%) + CsA48 (20%) + RA24 (20%), V: RE24 (20%) + CsE48 (10%), VI:RE24 (20%) + CmA72 (10%), VII: CsE48 (10%) + CmA72 (10%), VIII: CmA72 (10%) + CsE48 (10%) + RE24 (20%).

In summary, our results showed that the best treatments were obtained with concentrations of 2 mg/mL for *R. communis* and *C. bipinnatus* (100% nematodes immobility), while for *T. erecta* (1 mg/mL) the best results were obtained with acetone at 48 and 72 h of maceration, showing 100% immobility as well. These values were comparable to those obtained with the chemical nematicide (Verango), without showing significant differences between treatments, so they were considered the minimum inhibitory concentrations. Such treatments were combined 1:1 as shown in Figure 6. The combined treatments showed no evident synergy; only treatment III showed 80% immobility after 72 h of contact. Nevertheless, after washing, the immobility decreased to 70%; hence, this effect was considered both nematicidal and nematostatic.

### 2.4. Germination Index

**Figure 7:**
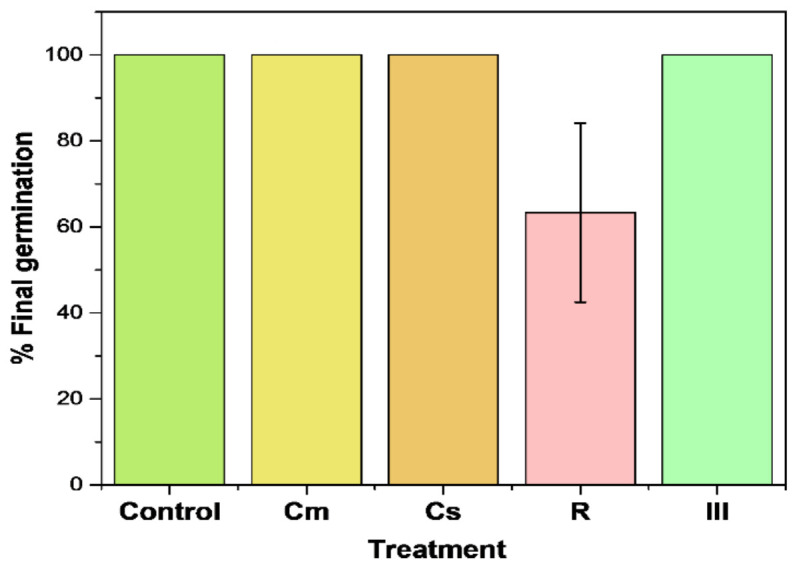
Final germination (%) of hybrid tomato seeds, V427, treated with different formulations of plant extracts. Control, Cm: extract of *T. erecta*, Cs: extract of *C. bipinnatus*, R: extract of *R. communis*, III: combination of extracts of Cm and Cs.

The germination index (GI) of hybrid tomato seeds V427 with different plant extracts was presented in Figure 7. For these analyses, the germination of 30 seeds of *Solanum lycopersicum* was considered for each treatment. In these experiments, only the best combination treatment was added (III: CmA48 (10%) + CsA48 (20%)), according to the results shown in Figure 6. To formulate the rest of the extracts, a 1:1 volume of each previously obtained extract from *C. bipinnatus* (Cs), *R. communis* (R), and *T. erecta* (Cm) was taken at the concentration of the highest nematicidal effect.

According to the ANOVA test (*p* = 0.05), the only significant difference between the extract treatments was shown by *R. communis*, with a final germination of 65%, after 12 days monitoring, while the rest of the treatments, including the control, presented a 100% final germination.

The GI is an indicator of the factors’ interactions that inhibit or promote germination in plants, as well as factors that promote the growing of roots [25]. Table 3 shows the average root growth of tomato seeds after treatment, with the different extracts compared to a control without treament. The ANOVA analysis for root and hypocotyl elongation did not show statistically significant differences between the extracts of *T. erecta* and *C. bipinnatus* as well as their combination. Nevertheless, the extract of *R. communis* was the only treatment where no hypocotyl growth was observed, which was considered a growth inhibitory factor where components such as rinin and ricinin could play a role.

**Figure 8:**
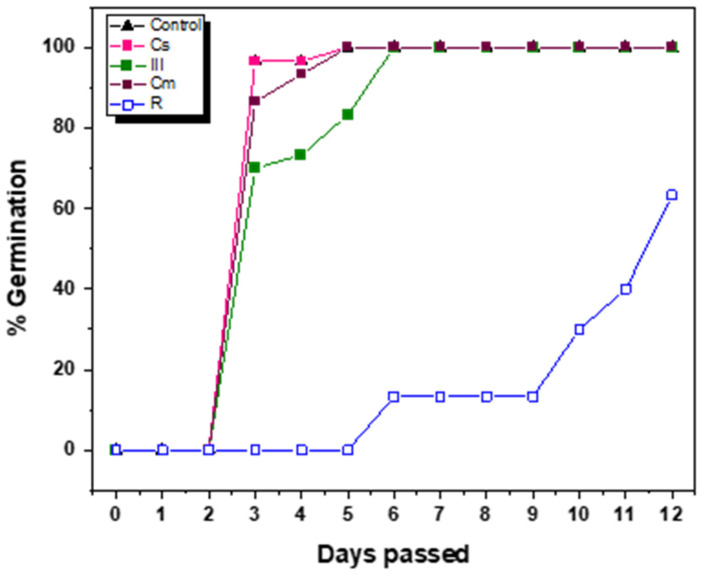
Germination rate of tomato seeds (%) influenced by the presence of plant extracts.

Figure 8 presents the effect of plant extracts on the germination rates over time. Treatments with extracts of *T. erecta*, *C. bipinnatus*, and combination (III) started germinating from day 3, as did the control, while the treatment with *R. communis* reached only 20% germination until day 6, and only achieved 60% by the end of day 12, when the rest of the treatments and control (overlapping with Cs treatment), reached 100% germination at day 6.

**Figure 9:**
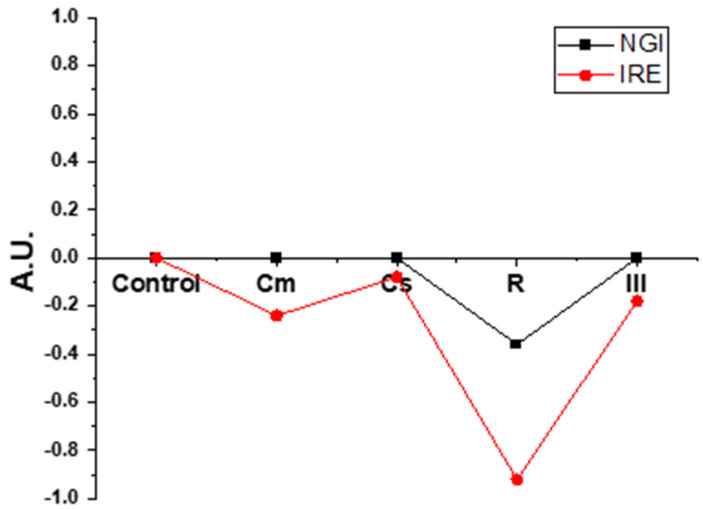
Normalized germination index (NGI) and Radicle Elongation (IRE) values of *Solanum lycopersicum* (−1 to +1) obtained from the bioassays with the different plant extracts.

To measure the toxicity rate of the germination index, we calculated the normalized residual germination index (NGI) and the normalized residual root elongation (IRE), which indicate a toxicity gradient with values from −1 to >0, where values from 0 to −0.25 mean low toxicity, −0.25 to −0.5 indicate moderate toxicity, −0.5 to −0.75 represent high toxicity, −0.75 to −1 mean very high toxicity, and >0 indicate root growth or hormesis [26]. Figure 9 shows the calculated NGI and IRE values obtained from the germination tests, where the control presents an NGI of 0, being considered as null toxicity as expected, and an IRE of 0, which indicates normal growth or hormesis of the radicle.

From the germination rate and root growth tests, the degree of toxicity of each tested extract on *S. lycopersicum* seeds was observed (according to the toxicity scale of Bagur et al., 2011) [25]. Thus, the treatments of *T. erecta*, *C. bipinnatus,* and III (a combination between *T. erecta* and *C. bipinnatus*), showed low toxicity according to their calculated NGI values; therefore, there was no inhibition of the germination rate. Similarly, the IRE values obtained for the three treatments indicate a low toxicity degree after contact with plant extracts, indicating that even when root growth is lower compared to the control, there is a normal germination rate without inhibition.

**Figure 10:**
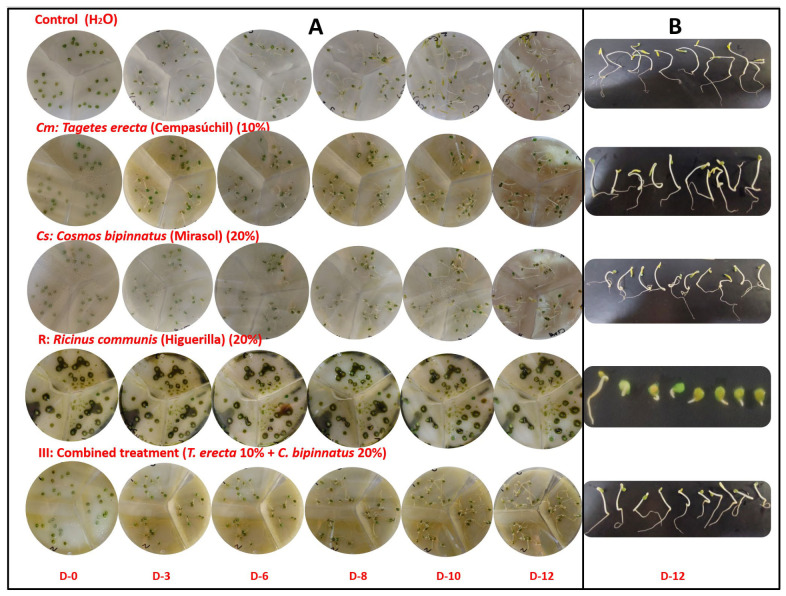
Germination tests with tomato seeds after contact with each plant extract. (**A**): Germination of *S. lycopersicum* seeds from 0 to 12 days. (**B**): Phenotypical analysis of root and hypocotyl elongation at 12 days after germination.

On the other hand, *R. communis* presented NGI values of −0.36, which indicates a moderate toxicity for seed germination, thus affecting the final germination rate. Also, the IRE value indicated a very high toxicity, which means that even when germination occurs, the radicle will not grow, as observed in Figure 10B, where the minimum growth of the radicle was observed in tomato seeds after 12 days of germination.

The treatment with *R. communis* showed a visible saturation of the extract around the seeds due to its oily nature, while the treatments with the other extracts had a suitable dispersion through the filter paper throughout the plate. In fact only 8 of 10 seeds can germinate up to 12 days after *R. communis* treatment. On Figure 10B, the elongation of roots and hypocotyl after contact with most of the treatments can be observed, confirming a low inhibitory effect from the applied extracts. However, a further evaluation after this phenological stage is still required to prove that plant development is not affected.

Other authors have reported the phytotoxicity effects of different plant extracts on seed germination. Duarte (2020) reported the toxicity of anise and rosemary essential oils which inhibited the germination of onion bulbs, affecting root elongation [27]. The oily nature of the extracts affected the cell division of the root system, due to the presence of allelochemicals causing an abnormal growth effect. The emerged roots presented only 3 mm at most, while the control showed root elongation up to 3.5 cm. Furthermore, it significantly reduced germination by up to 40%, below the control and the other treatments [27,28,29], just as we observed for the *R. communis* treatment (Figure 10). Moreover, Wang et al. (2022), mentioned that the phytotoxic effects observed in seeds affect subsequent vegetative development, since cell division is not properly accomplished, with prolonged exposure to allelopathic compounds causing root inhibition, as observed for the treatment with *R. communis* [30]. Furthermore, once root growth was inhibited, there was null hypocotyl development (Figure 10B). Therefore, this treatment would not be recommended as a preventive treatment during germination. However, it should not be ruled out as a possible herbicide or fumigant in later stages of vegetative and fruit development, where the plant has a complete biological system capable to combat and even use metabolites present in plant extracts [30,31].

### 2.5. Evaluation of Plant Extracts Under Greenhouse Conditions

**Figure 11:**
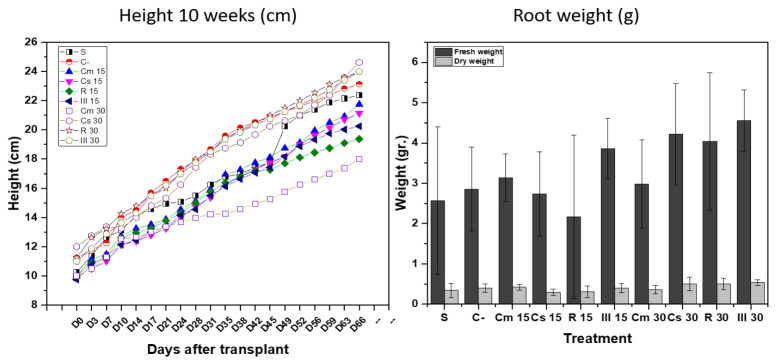
Height and root weight monitored over 10 weeks after treatment with plant extracts Cm, Cs, R, and III, and controls S and C-, inoculated every 15 and 30 days.

A first cycle of treatments was established in a greenhouse crop, using four pots for each plant extract treatment (Cm, Cs, R, and III) and the same pot number for controls (S and C-), inoculated every 15 and 30 days. Phenotypic monitoring was carried out by taking pictures twice a week, to monitor the morphological changes in plants observed over 10 weeks of treatment, as presented in Figure S2: Phenological analysis of the development of tomato plants treated with plant extracts Cs, Cm, R, and III; cycle I (Supplementary Material). In general, the observed phenological characteristics do not present differences related to aerial symptoms until week 8, shown by plants affected by nematodes, such as chlorosis or wilting of leaves. However, the R treatment based on the *R. communis* extract presented leaf curling, which was an indication of stress in the plants due to lack of macronutrients, water stress, or the presence of phytotoxic residues [32], which coincides with the previous results. Since the *R. communis* extract was the only treatment that induced these changes in tomato plants, it is likely that such effects were caused by the presence of phytotoxic metabolites that affect plant growth and development. Figure 11 showed the effects of all treatments with respect to the height and root development of tomato plants, where the R treatment inoculated every 15 days showed the greatest effect on roots. Treatments based on Cm, Cs, and III do not present statistically significant differences between those inoculated every 15 days with respect to the controls, both in the height and fresh and dry weight of roots. Conversely, there was a notable difference compared to treatments every 30 days with the plant extracts, from which treatment III (combination of *T. erecta* and *C. bipinnatus* extracts) showed the greatest height and root development (fresh mass), followed by Cs treatment (*C. bipinnatus* extract treatment).

**Figure 12:**
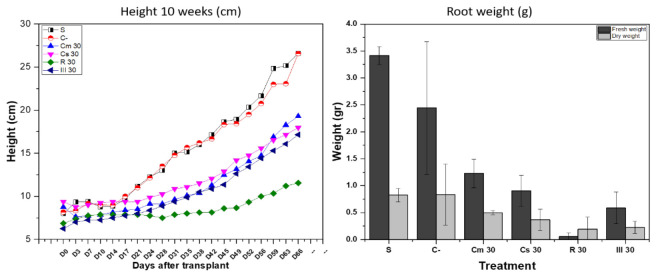
Height and root weight monitored over 10 weeks after treatment with plant extracts Cm, Cs, R, and III, and controls S and C-, inoculated every 30 days.

Based on the results obtained from the first cycle, the experiment was repeated almost under the same conditions (four pots for treatment), now discarding the treatments inoculated every 15 days (cycle II), leaving just the inoculations before transplantation as a preventive treatment, also 30 and 60 days after transplantation. Similarly, the phenotypic analysis of cycle II did not show aerial symptoms related to the presence of nematode galls, and unlike the first cycle, treatment R did not cause leaf curling on plants, as observed in Figure S3: Phenological analysis of the development of tomato plants treated with plant extracts Cs, Cm, R, and III; cycle II (Supplementary Material). Figure 12 shows the increase in height and root development over 10 weeks. In general, the heights obtained in this experiment were lower than those observed in the first cycle, probably due to the different seasons when growth experiments were performed (spring for cycle I and winter for cycle II). As observed before, treatment R caused effects on tomato plants related to the lowest heights and root weight (fresh mass). For the case of Cm, Cs, and III treatments, non-significant differences were found; however, heights were lower than that of controls (S, C-). This lagged behavior could be explained by the application of plant extracts, which present a slight toxicity index in seeds, as observed in the germination test (especially in R treatments), which could also be related to the stem development of plants, but the toxicity is not high enough to prevent plant growth.

**Figure 13:**
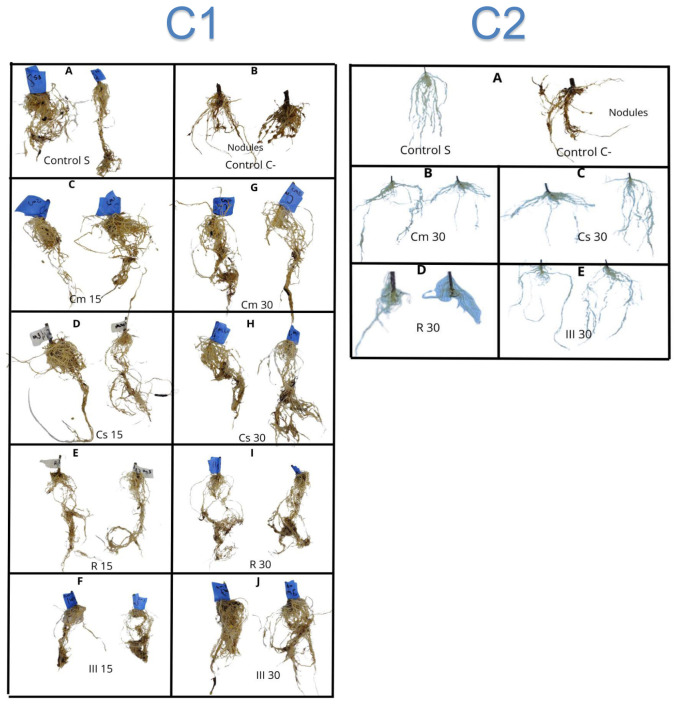
Morphology of tomato roots at the end of treatments from cycles 1 and 2. C1: First crop cycle evaluated. A: Control S, B: Control-, C: Treatments every 15 and 30 days with addition of plant extract. C2: Second crop cycle evaluated. A: Control S and Control-, B: Treatments with addition of extract every 30 days.

Figure 13 shows the morphology of the plant roots from each of the treatments evaluated during cycles I and II, indicated as C1 and C2 in Figure. According to the root infection scale proposed by Taylor and Sasser (1983) [33], it was found that the treatments with plant extracts every 15 or 30 days had 0% infection, since all of the roots were clean and free of galls, compared with control S (Figure 13(C1-A),(C2-A)), while control C- was between 1 and 11% on the infection scale (Figure 13(C1-B),(C2-B)). It was perceived that the root development was lower for cycle II compared to the first cycle, which reduced the height of roots over the weeks. In addition, secondary roots were responsible for the uptake of water and nutrients. This observation could explain that deficient root development causes a decreased uptake of water and nutrients, consequently affecting plant growth. It is suggested that such effects probably result from the decrease in the photosynthetic activity and vegetative stage of plants, characteristic of the winter seasons [34].

### 2.6. Characterization of Plant Extracts

The Folin–Ciocalteu method was used to characterize the plant extracts, by measuring total phenols and flavonoids. The results were expressed as mg of gallic acid from a calibration curve for the quantification of total phenols, and a quercetin curve for the quantification of total flavonoids in the case of the three plant extracts (Table 4).

The ketone extract of Cm had a lower quantity of phenols than that reported by Monroy (2023), finding values between 25 and 80 mg of GA/g and 20 to 38 mg of EQ/g from the extract [35]. However, it should be noted that these results were obtained from the flowers of *T. erecta*, which contains a high quantity of these compounds in flowers, leaves, and fruits. Therefore, in our work a lower quantity of both compounds was obtained, since roots were the only part of the plant used to prepare the extracts. In the case of *C. bipinnatus* plant, there are reports of ketone and aqueous extracts obtained from the petals of the purple flower reaching values of 45.5 mg EQ/g and 34.46 mg GA/g from the extract [36,37]. In this study, the measured values of phenols and flavonoids were lower than previously reported, since the extracts were obtained from stems, leaves, and whole flowers, reducing the concentration of these metabolites found only in the flowers with purple or pink coloration, which contain a higher concentration of anthocyanins. For *R. communis,* Surco-Laos F. et al. (2022) reported the phenol and flavonoid content from ethanolic and hydroethanolic extracts prepared from leaves, obtaining 20.9 mg of GA/g and 0.99 mg of EQ/g from the extract [38]. The values reached in this work are close to those reported, considering that the extract was obtained from the seeds of *R. communis*.

The chemical composition of the ketone extracts was analyzed by high-performance liquid chromatography (HPLC). Table 5 shows the main components identified for each of the plant extracts.

Using standard references, the major compounds present in the analyzed plant extracts were mostly identified. A total of 35 components were found, some of which are related to polyphenols such as ellagic acid and apigenin present in *T. erecta*, which are associated with compounds with antimicrobial, antifungal, and antiparasitic properties [39,40]. Although the presence of thiophene and its derivatives was expected, such compounds were not found in this analysis, even though several authors have reported their major presence in *T. erecta* roots, observed at wavelengths of 330 nm [41]. Since our identification method was performed at 250, 254, and 280 nm, this could explain that thiophene compounds were not detected. For the case of *C. bipinnatus*, a total of 55 components were found, with catechin and benzoic acid being the most frequently identified. These compounds are mainly reported for their anti-stress function, especially as reaction mechanisms against free radicals also attributed to antifungal and antimicrobial properties [42,43].

Lastly, *R. communis* presented a total of 30 compounds, with ricinoleic acid, quercetin, and gentisic acid predominating. Ricinoleic acid has been one of the most studied components from *R. communis,* because it is majorly present in the seeds, from which commercial castor oil is obtained with proven effects against pathogens. Moreover, the presence of ricinoleic acid is indicative of ricin and ricinin metabolite content, which are considered highly toxic to living organisms, but possess antimicrobial, antifungal, and insecticidal properties [44].

## 3. Discussion

PPN are among the most important phytoparasites of concern, which infect tomato crops in Mexico and many other countries [45]. Tlaxcala is one of the tomato producing states, where the presence of these nematodes has been reported. A tomato greenhouse was sampled at five random diagonal points to identify phytoparasites, from which nematode species were molecularly identified as *M. incognita* and *N. aberrans*, by a proportion between 20% and 80%, respectively.

It is noticeable that only few works report the presence of this class of nematodes in Tlaxcala region. For instance, del Prado et al. (2018) showed the analysis of 56 points in Mexico, identifying *M. incognita*, *M. arenaria*, and *M. hapla* nematodes in potato cultivars from Tlaxcala [46]. Likewise, Flores et al. (2008), reported the presence of *N. aberrans* in tomato, chili, and bean crops in Tlaxcala [47]. The presence of both nematodes (*Meloidogyne* sp. and *N. aberrans*) in the same crop has been described by some authors, describing that *Nacobbus aberrans* is capable of displacing different species of *Meloidogyne* spp., showing better resistance to environmental stress conditions compared to *Meloidogyne* spp. [47]. This coincides with the results obtained from our sampling points within the greenhouse. For instance, the samples taken near the water irrigation tanks that have constant dispersion of water showed major presence of *M. incognita*. The rest of the greenhouse samples coincided with the presence of *N. aberrans*, which does not produce the same humidity, and it is easy for *N. aberrans* to displace *M. incognita*, adapting to lower humidity and higher temperature. These results agree with Lax et al. (2022), where it is stated that *Nacobbus* spp. can be found in very different environments due to its great adaptive capacity [48]. Furthermore, Velloso et al. (2022), found that three species of *Meloidogyne* (*incognita, enterolobii*, and *floridensis*) were more aggressive at higher temperatures [49].

On the other hand, Rafiq et al. (2025) mention that the *Asteraceae* plant family has several pharmacological properties, as well as its allelopathic potential for combating plant pathogens, associated with components such as phenolic acids, sterols, and polysaccharides, among others [50]. Some plants within this family are considered weeds, which justifies their use, as is the case with *C. bipinnatus*. Since *Tagetes erecta*, *Cosmos bipinnatus,* and *Ricinus communis* belong to the Asteraceae family, some of their bioactive compounds are capable of immobilizing some genera of PPN [51,52].

The in vitro experiments carried out in this work demonstrated the effect of the plant extracts obtained from these three plants using different solvents, which can improve the selectivity of secondary metabolites. Acetone was established as the best solvent for obtaining phenolic and polyphenolic compounds with antimicrobial, antiviral, and insecticidal effects, and in our case nematicidal effects [50]. The extracts obtained with hexane did not show a nematode immobility effect higher than 60%. It has been reported that these types of extracts can contain sterols and saponins [53], which could provide antimicrobial and antifungal effects; however, this has not been commonly associated with PPN.

Treatments with plant extracts macerated with ethanol and acetone showed a 100% immobility effect starting from 2 mg/mL concentration, decreasing their effectiveness when the extract concentration was reduced to 1 and 0.5 mg/mL. The best results show that 100% nematode immobility was obtained for *T. erecta* (1 mg/mL) macerated with acetone, and with *C. bipinnatus* and *R. communis* both at 2 mg/mL with acetone and ethanol. Thus, treatments labeled RA24, CmA48, CsA48, RE24, CsE48, RE24, and CmA72 were chosen to generate combinations, evaluating the synergism between extracts. However, the highest immobility obtained from one combination of CmA48 and CsA48 was 80%, marked as the best combined treatment (III), as observed in Figure 6. It is suggested that the rest of the combinations showed antagonism between their components, decreasing the nematicidal effect observed when used individually. In general, the resulting mixture of extracts with antimicrobial or antifungal effects depends on the composition and concentration present in each extract. Synergy exists when the addition of these components increases the antimicrobial, antifungal or nematicidal activities, either by the chemical interaction between the compounds, modifying the selectivity or potential of the membranes or modifying some proteins or receptors. Conversely, antagonism occurs when the availability of metabolites present in the extracts depends largely on the type of solvent and polarity [54,55]; however, there is scarce information about the combination of extracts, and further studies are needed to find the ideal synergism between two or more compounds present in total extracts.

Accordingly, the characterization of our plant extracts allowed for the identification of the major compounds, which coincided with the previous determination of total phenols and flavonoids. Phenolic compounds are consistent with those reported in the literature, and the presence of several polyphenols was confirmed by HPLC. One of these identified compounds is the ricinoleic acid, related to the presence of plant albumins such as ricin and ricinin, considered highly toxic to living organisms (see Table 5). Ricin is the main component of the *R. communis* plant, mainly present in seeds or fruits, and classified as the most toxic ribosome-inactivating protein type 2 (RIP) [56,57]. This is a non-polar component; however, when using solvents such as ethanol, there may be better interaction with other polar components that can be carried away by this solvent, as was observed in treatment VIII. On the other hand, for treatment IV, a lower nematicidal effect was obtained. Since these extracts were obtained from the three plants with acetone, it is possible that a medium polarity can cause weak interaction between the components when *R. communis* was present, since this plant has mostly hydrophobic compounds [58]. Ricin is made up of two polypeptide chains: one capable of inhibiting protein synthesis, and the other capable of binding to carbohydrates present in cell membranes [32]. These features can be responsible for affecting the seeds and plants of *S. lycopersicum*, since once they inactivate the ribosomes, the functions that activate the germination process or the development of new tissues are lost. This is correlated with our observations during the germination tests, and the lower development of roots and foliage in the treatments containing *R. communis* extracts. The use of pure ricin is highly regulated in Europe, due to its high toxicity and danger caused to fauna, humans or even microbial life [59,60]. Nevertheless, there are no reports on crude extracts that contain ricin, so the effects in this field remain unknown. One study has reported on the use of biomass obtained from *R. communis* seeds, which contain ricin as their main component, reporting benefits such as an accumulation of high amounts of nutrients such as N, P, and K, promoting microbial activity in soils [61]. Therefore, it is necessary that the different components present within the *R. communis* extracts, used in this study, are further characterized in productive soils before recommending their use.

Regarding the nematicidal activity of the treatments under controlled conditions, the phenotypic analysis does not show major differences in either of the two evaluated cycles, within the first 10 weeks after transplantation. The development of the plants was normal, beginning typical differentiation in height between 14 and 17 days, where the adaptation time after transplantation must be considered [25].

From the first cycle, it was observed that the crops treated with plant extracts every 15 days showed lower heights than those treated every 30 days, correlating our results with those obtained from the germination tests, where a prolonged time with plant extracts inhibited the growth of hypocotyl seeds compared to the control. In addition, it was possible that a greater amount of plant extract treatment on the tomato crops could alter their growth. Treatments with a longer period between the inoculation time (every 30 days) reflected the greatest growth on plants, comparable with the C- and S controls; but, most importantly, null galls were observed in the roots at the end of the treatment period. For the second cycle, the same behavior was repeated in height and absence of galls on the roots. The treatment with *R. communis* also presented the lowest height, with castor oil as the main metabolite that could cause this phytotoxic effect not only on the nematode, but also on the development of the plants. This could also be observed in the development of the roots (fresh and dry weight), where the Cs, Cm, and III treatments showed the highest amount of fresh mass. This is due to the induced development of secondary roots and root hairs, which were mainly responsible for the absorption of water and nutrients, as well as interaction with other organisms, including pathogens and symbiotic microorganisms. This could be attributed to better plant development, as expected, where phytoparasites are not present and do not compete for nutrients, and therefore, higher yields can be obtained in the productive stages [48]. At the same time, the dry mass decreases, since the plants were mostly made up of water that is lost when humidity is removed; thus, no significant differences were found in dry weights.

The repeated experiment in greenhouse crops enabled us to corroborate that the treatments performed every 30 days with plant extracts were suitable for inhibiting the two species of nematodes found in this study, which cause galls in tomato roots.

It is worth mentioning that null nodules were found in the roots of tomato plants, except in the C- controls; hence, treatment every 30 days with the extracts Cs, Cm, R, and III was enough to control the nematode population without causing a negative effect on the development of tomato plants. Conclusively, it would be expected that in productive soils, our results could improve interaction with nutrients and beneficial microorganisms that help in the growth of plants as a synergistic effect between extracts, plants, and soil.

## 4. Materials and Methods

### 4.1. Molecular Identification of the Nematodes

#### 4.1.1. Sampling

Tomato roots were collected from a greenhouse producing the tomato hybrid V427, affected by nematodes, from a community located in Tlaxcala, Mexico, at 19°17′52.7″ N 98°15′42.2″ W. Diagonal random sampling was established from 5 different points in the greenhouse, obtaining roots with notable gall formation in six-month-old plants.

#### 4.1.2. Nematode Isolation

Female nematodes with morphological differences were isolated from the collected roots by direct dissection of the galls, using a microscope and dissection equipment. The collected nematodes were rinsed and placed in Eppendorf tubes (3 to 5 per tube) for lyophilization [62], yielding a total of ten isolates.

#### 4.1.3. DNA Extraction

The DNeasy^®^ Blood & Tissue kit was used following the manufacturer´s (QIAGEN, MD, USA) for DNA extraction, which was stored at −20 °C. The Polymerase Chain Reactions (PCRs) were set using the universal primers to identify the phylum Nematoda, which are the following:

Nem18sF: 5′-CGCGAATRGCTCATTACAACAGC-3′;

Nem18sR: 5′-GGGCGGTATCTGATCGCC-3′.

The PCR conditions were modified as follows: 1 cycle at 94 °C for 5 min, 37 cycles [94 °C for 45 s, 56.2 °C for 45 s and 72 °C for 1.5 min], and 1 cycle at 72 °C for 10 min [22]. Finally, the DNA samples were purified with the DNA Clean & Concentrator^®^-5 kit (ZYMO RESEARCH, CA, USA) and sent for sequencing. The received sequences were analyzed and registered on the NCBI platform.

### 4.2. Molecular Identification of Plants and Preparation of Plant Extracts

#### 4.2.1. Collection of Plants

Different leaf tissues were collected from three plant species commonly known as higuerilla (*Ricinus communis*), cempasuchil (*Tagetes erecta*), and mirasol (*Cosmos bipinnatus*). The collected tissues from each species are described in Table 6, as well as the solvents used to prepare the plant extracts.

#### 4.2.2. Molecular Identification of Collected Plants

Molecular identification was carried out by collecting young leaf tissues from the three plants, which were lyophilized in a LABCONCO system for 24 h. DNA extraction from plant tissues was performed by the CTAB method, as follows: 500 μL of cetyltrimethylammonium bromide (CTAB) and 0.3% β-mercaptoethanol were added per 20 mg of leaf sample, heating the mixture at 65 °C for 30 min, and then centrifuged at 500 rpm for 10 min, and the supernatant was removed. Chloroform was added to the samples and mixed by immersion for 5 min, then centrifuged at 11,000 rpm for 10 min. The supernatant was recovered, and isopropanol was added. Then, the sample was centrifuged at 15,000 rpm for 5 min, and the pellet was recovered by washing three times with 70% ethanol and water, and then incubated for 30 min at 37 °C. Extracted DNA was stored at −20 °C [63]. For the PCR setup, two reported primer pairs were used as described by Naim et al., (2020) [24]:

rbcL-F: 5′-ATGTCACCACAAACAGAGACTAAAGC-3′,

rbcL-R: 5′-GTAAAATCAAGTCCACCRCG-3′

rpoC1-F: 5′-GGCAAAGAGGGAAGATTTCG-3′

rpoC1-R: 5′-CCATAAGCATATCTTGAGTTGG-3′

PCR was performed under the following conditions: 1 cycle at 94 °C for 5 min, 35 cycles [94 °C for 45 s, 53 °C for 45 s, 72 °C for 40 s] and one cycle at 72 °C for 10 min. The extracted DNA was purified using the commercial kit DNA Clean & Concentrator TM-5 (Zymo Research, CA, USA). Samples were sent for sequencing to the National Laboratory of Agricultural, Medical, and Environmental Biotechnology (LANBAMA) at IPICYT San Luis Potosi, Mexico. The sequences were analyzed using the bioinformatics software Geneious Prime (Free online version), performing alignments with the species mentioned in Table 6.

#### 4.2.3. Preparation of Plant Extracts

The collected plant tissues were dried at room temperature for 15 days, or until moisture was completely removed before pulverization. Plant tissues were selected according to the existing reports about nematicidal effects, associated with the presence of specific metabolites. For every 100 g of plant, 400 mL of each reagent-grade solvent (*w*/*v*) was added (as shown in Table 6), according to the modified methodology of Rafiq et al. (2024) [64].

Maceration was performed for 24, 48, and 72 h, with each solvent at room temperature, aiming to obtain the highest amount of flavonoids and phenolic compounds, using acetone, ethanol, and hexane [65]. Each extract was filtered twice through Whatman filter paper #4 and #1, respectively. The filtrate was concentrated in a rotary evaporator at 35 °C, under reduced pressure until dry. To calculate the obtained yields of plant extracts, amber vials were washed and dried to a constant weight. The yields were calculated using Equation (1), where Wf = final weight of the vial with extract, Wi = initial weight of the dry vial, and Ws = used weight of the pulverized plant. The dry extract was stored at 4 °C, protected from light [66]. The experiments were repeated five times in triplicate to calculate the yield of each plant per 100 g.

(1) %Yield=((Wf−Wi)/Ws)100

### 4.3. Nematicidal Activity

Egg masses and J2 were extracted from tomato roots affected by nematodes, without discriminating species. Sampling was conducted in production greenhouses from Tlaxcala, Mexico. The extraction method used was maceration–sieve filtering [65,66]. The extracted nematodes were incubated at 25 °C for 3 days.

#### 4.3.1. In Vitro Testing of Plants Extracts

The plant extracts were initially prepared at 2 mg/mL concentration, as reported by Arboleda et al. (2012) [20]. For *R. communis* extracts, the minimum concentration for activity on phytoparasites was 20%, equivalent to that suggested in this work. The extracts of *T. erecta* and *C. bipinnatus* were prepared at the same concentration. Fluopyram (Verango, Bayer) was used as a chemical control dissolved in distilled water at 50 mg/mL. As a negative control, a 10% Tween 20 solution dissolved in distilled water was used.

For each treatment, ten J2 individuals were added in 24-well plates, adding 100 µL of each solution (including treatments and controls) to determinate the effect of plant extract treatment in PPN (present in productive greenhouses in Mexico) that cause root gall formation in tomato plants. The immobility percentage responses were recorded every 24, 48, and 72 h, and each J2 was stimulated to verify if it was dead, where null movement after stimulation was considered dead.

After 72 h, the J2 were washed in a 400 mesh sieve to remove excess extracts or testing substances, then recovered and placed in Eppendorf tubes with 1 mL of distilled water, and checked 24 h after washing. If the nematodes remained immobile, they were considered dead and the effect was considered nematicidal; if any individual recovered its mobility 24 h after washing, the effect was considered nematostatic [1]. The procedure was repeated with all treatments, but now with concentrations of 5% and 10% for each extract, and each experiment was repeated twice by triplicate. The immobility percentage was calculated using Equation (2), where i = immobility percentage, nt = active individuals (J2) in the treatment, and nc = active individuals (J2) in the control [67].

(2) $$i=\left(1-\frac{nt}{nc}\right)100$$

#### 4.3.2. Phytotoxicity Test

A germination test was carried out with 30 seeds per extract [26] of *Solanum lycopersicum* variety Saladet hybrid V427. The extracts were dissolved in a 10% Tween 20 solution with distilled water, and the same solution was used as a control at 10% Tween 20 without plant extract [30]. Monitoring was carried out for 14 days, considering the experimental variables final germination (%FG), germination rate (%), radicle length (cm), and hypocotyl length (cm), as well as the normalized residual germination index (NGI) and the normalized residual root elongation (IRE) with Equations (3)–(5) [26].

(3) $$\%FG=\frac{Number\;of\;germinated}{Number\;of\;totalseeds}*100$$

(4) $$NGI=\frac{Gx-Gt}{Gt}$$

(5) $$IRE=\frac{Ex-Et}{Et}$$

where *Gx* = average percentage of germinated seeds with extract, *Gt* = percentage of control germinated seeds, *Ex* = average length of the radicle from germinated seeds with extract, *Et* = average length of the radicle from germinated seeds in the control [26].

### 4.4. Evaluation of Plant Extracts in Greenhouses

Forty certified tomato seeds of the V427 hybrid were germinated in an inert substrate, generated with peat moss and perlite at a ratio of 1:3 (Peat moss Black Bale Lambert Canada, Multiperl Hoticultural perlite Floramundo Laguna Mills, CDMX, Mexico). Three days before transplanting, each seedling was immersed in a previously prepared solution of plant extracts, with four seeds for each plant extract treatment (the best selected from in vitro tests) at the different concentrations, as a preventive treatment. In the case of controls, the plants were immersed only in distilled water. The seedlings were transplanted into 10 L pots (four for each plant extract treatment and the same pot number for controls) with the inert substrate (peat moss/perlite 1:3), simultaneously inoculating them with a minimum of 100 nematodes per seedling, excluding the negative control, as close to the plant’s main root as possible. Plant height was monitored by treatment over a 10-week period according to a previous work, where the nematode effect in tomato plants in pots was observed within the first 2 months after transplantation [66].

Plant extracts were added every 15 and 30 days after transplanting, according to the nematode life cycle [66]. At the end of the treatments, the root of each plant was extracted to observe possible gall formation, and for comparison using the Taylor–Sasser scale based on the percentage of root gall formation.

### 4.5. Chemical Characterization of Plant Extracts

The total amount of phenolic compounds was quantified using the Folin–Ciocalteu method. For this method, 0.500 g of dry extract was resuspended in 5 mL of ethanol, stirred for one hour, and then filtered to remove impurities. Then, 200 µL of standard solution, and alternatively 200 µL of sample, were used (both prepared with this solution). Finally, 900 µL of Folin reagent and Na_2_CO_3_ was added, and the mixture was incubated for 90 min, and quantification was performed using a UV/Vis spectrophotometer at 750 nm (adapted from Zugazua et al., 2024) [68].

For the quantification of flavonoids, a modification of the colorimetric technique with aluminum trichloride, as reported by Ramos et al. (2017), was implemented [69].

In addition, a standard curve was prepared using 50 µL of a quercetin solution and 50 µL of the tested samples. Finally, the absorbance was measured in a UV/Vis spectrophotometer at 410 nm [69].

For HPLC quantification, a mixture of acetonitrile (ACN) and acidified water (AW) was used as the mobile phase, and the specific conditions are shown in Table 7. For separation, an Eclipse XDB-C18 column (ZORBAX) (4.6 × 250 mm, 5 μm) was used as the stationary phase, with 1 mL/min reversed-phase injection. A volume of 20 μL of each sample was injected into an Infinitely Better 1200 HPLC system at 250, 254, and 280 nm.

### 4.6. Statistical Analysis

All data obtained from the experiments and their corresponding replicates were subjected to analysis of variance (ANOVA) using OriginPro 2021 software, as well as the Tukey HSD multiple comparison test, with a confidence level of 0.05%. The data used were obtained from each experiment with at least three replicates (Table S1: Immobility of plant-parasitic nematode experimental data, Supplementary Material).

## 5. Conclusions

PPN affecting greenhouse tomato crops from Tlaxcala, Mexico, were molecularly identified as *Meloidogyne incognita* and *Nacobbus aberrans*, both nematodes present in the same crops. In our results, we demonstrated the nematicidal and nematostatic activities of plant extracts obtained from three species, *Ricinus communis*, *Cosmos bipinnatus*, and *Tagetes erecta*, proving their effectiveness against both identified nematodes. In addition, the phytotoxic effect of the extracts was evaluated on the growth of the radicle and hypocotyl of tomato seeds, where only castor oil from *Ricinus communis* presented high toxicity with respect to the tomato plant. Despite this, it does not inhibit growth; however, further studies are needed to spread their use in plant extracts.

Furthermore, the extracts of the three plants were tested in a greenhouse crop, inoculating the plants every 15 and 30 days in the first cycle. The development of tomato plants was followed over 10 weeks, demonstrating the efficacy of the three plant extracts against the nematodes present in crops. From these results, it was concluded that inoculation every 30 days was enough to control and inhibit the development of both nematodes, highlighting that this result was also obtained in the second cycle.

Finally, the chemical compounds identified by HPLC showed the abundance of phenolic components present in the analyzed plant extracts, which are consistent with the literature and provide a nematicidal effect. Further research will enable us to propose alternative bioproducts based on plant extracts that provide improved advantages of minimal environmental impact and high yields of organic crops.

## Figures and Tables

**Table 1:** Nematode molecular identification from tomato roots isolates.

Isolated	NCBI Code	Bit Score	Genus and Species	% Identity
P1A2	AF442190	1417.5	*Nacobbus aberrans*	100
P2A2	KT591482	771.173	*Nacobbus aberrans*	99.5
P2A3	KT591480	1417.5	*Nacobbus aberrans*	100
P3A2	KT591481	1415.65	*Nacobbus aberrans*	99.9
P3A3	KT591483	776.713	*Nacobbus aberrans*	99.8
P4A2	JX100421	1319.63	*Meloidogyne incognita*	99.8
P4A3	ON123406	1005.7	*Meloidogyne incognita*	99.6
P5A1	AJ966494	1312.24	*Nacobbus aberrans*	99.6

**Table 2:** Molecular identification of the three collected Higuerilla, Cempasuchil, and Mirasol plants.

Primers	Isolated	Identification	% Identity
rbcL	H2BCRBC	*Ricinus communis*	99.64
rpoc	H1PO	*Ricinus communis*	99.64
rbcL	S2BC	*Cosmos bipinnatus*	99.46
rpoc	S1PO	*Cosmos bipinnatus*	100
rbcL	MIBC	*Tagetes erecta*	99.77
rpoc	MIPO	Unidentified	Unidentified

**Table 3:** Effect of different extracts on the lengths of the radicle and hypocotyl of tomato seeds.

Treatment	Root (cm)	Hypocotyl (cm)
Control	3.43667 ± 1.44 ^a^	3.88333 ± 1.27 ^a^
Cm	2.60333 ± 0.32 ^ab^	1.94333 ± 0.41 ^b^
Cs	3.13667 ± 0.94 ^ab^	2.05333 ± 0.50 ^b^
R	0.18167± 0.24 ^c^	0.0 ± 0.0 ^c^
III	2.81667 ± 0.58 ^b^	1.53333 ± 0.47 ^b^

Means showing the same letter along the columns indicate no significant difference at *p* ≤ 0.05.

**Table 4:** Quantification of total phenols and flavonoids from three plant extracts.

Plant Extract	Source Plant	Total Phenols (mg GA/g Extract)	Flavonoids (mgQ/g Extract)
Cm	*T. erecta*	13.52 ± 0.01	0.6 ± 0.1
Cs	*C. bipinnatus*	17.42 ± 0.01	6.90 ± 0.1
R	*R. communis*	18.889 ± 0.01	0.013 ± 0.05

**Table 5:** Main chemical components of ketone extracts identified by HPLC.

No.	Plant	Tissue	* RT (min)	Wavelength (nm)	Abundance (%)	Possible Compound
1	*T. erecta*	Root	2.447	250, 254	0.77	Gallic acid
2			3.280	250	0.49	Protocatechus acid
3			3.700	250, 280	0.39	Catechine
4			4.713	250, 254	1.70	Epicatechin
5			6.687	250, 254	19.45	Ellagic acid
6			7.100	250, 254, 280	6.54	Unidentified
7			8.347	250, 280	3.38	Unidentified
8			9.860	250, 254	6.54	Unidentified
9			11.567	250,254,280	2.43	Β-mircene
10			12.767	250, 254	1.45	Floridzine
11			15.513	250, 254, 280	1.24	Terpinolene
12			15.780	250	0.71	Quercetin
13			18.013	250	6.98	Unidentified
14			20.333	250, 254, 280	41.20	Apigenin
1	*C. bipinnatus*	Flower, leaves, and stem	2.56	250, 280	0.18	Gallic acid
2			3.140	280	2.80	Phenylalanine
3			3.880	254,280	17.07	Catechine
4			4.173	254, 280	52.30	Benzoic acid
5			4.827	250, 254, 280	2.70	Epicatechin
6			5.627	280	0.59	Routine
7			6.120	254, 280	4.90	Ellagic acid
8			6.627	254, 280	1.84	2-hexanal
9			10.920	280	8.92	Unidentified
10			15.460	250, 254, 280	0.08	Quercetin
1	*R. communis*	Fruit/seed	2.453	250, 254, 280	1.80	Gallic acid
2			2.547	280	1.37	Syringic acid
3			3.113	280	0.13	Caffeic acid
4			3.947	280	0.36	Catechine
5			4.173	250, 254, 280	63.42	Ricinoleic acid
6			5.133	250, 280	1.64	Routine
7			8.920	280	1.56	Unidentified
8			10.300	250, 254	6.61	Gentisic acid
9			17.36	250, 280	1.21	1,2,3,6-Tetragalloylglucose
10			19.133	280	28.32	Quercetin
11			22.067	250, 280	0.55	Naringenin

* RT: retention time.

**Table 6:** Plants used for extraction experiments.

Type of Plant	Collection Site	Organs of the Plant Used	Extraction Solvent
Higuerilla	19°18′58″ N 98°16′12″ W	Whole seed	Hexane, Acetone, Ethanol
Mirasol	19°17′00″ N 98°17′00″ W	Leaves, stem, flower	Hexane, Acetone, Ethanol
Cempasuchil	19.229° N 98.218° W	Root	Hexane, Acetone, Ethanol

**Table 7:** Dilution gradients used for HPLC.

Time (min)	ACN%	WA%
0	20	80
5	25	75
10	30	70
15	40	60
20	60	40
25	30	70
30	20	80

## Data Availability

The data presented in this study are available on request from the corresponding author.

## Supplementary Materials

The following supporting information can be downloaded at https://www.mdpi.com/article/10.3390/plants15121872/s1, Figure S1: Sequences obtained from DNA sequencing of three plants; Figure S2: Images taken for phenological analysis of the development of tomato plants treated with plant extracts CS, Cm, R, and III. Cycle I (C1); Figure S3: Images taken for phenological analysis of the development of tomato plants treated with plant extracts CS, Cm, R, and III. Cycle II (C2); Table S1: Immobility of plant-parasitic nematode experimental data.
