# Supplementary figures and images for: Evaluation of the Nematicidal and Phytotoxic Potential of Ricinus communis, Cosmos bipinnatus, and Tagetes erecta Plant Extracts in Tomato
Source: Plants (Basel). 2026 Jun 17;15(12):1872. doi: 10.3390/plants15121872 (PMC13306631; doi:10.3390/plants15121872)

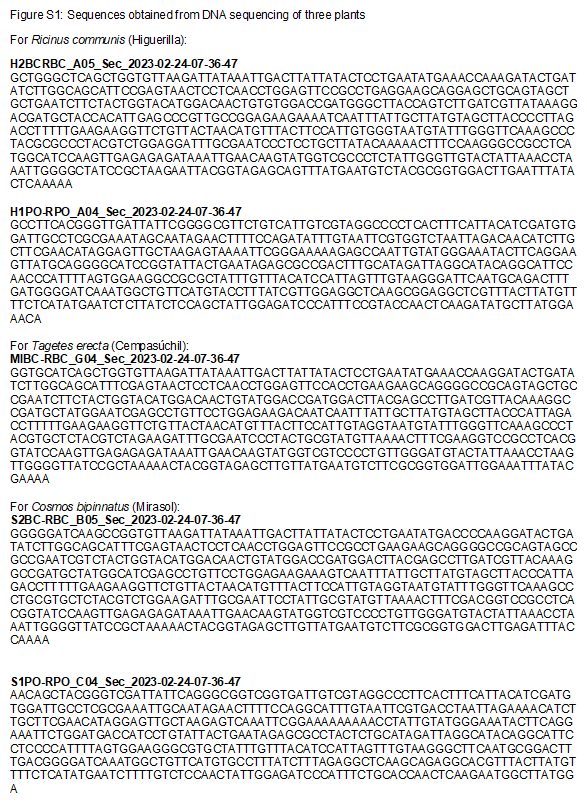

Supplement: Supplementary file 1 [file plants-15-01872-s001.zip › Figure S1.png]

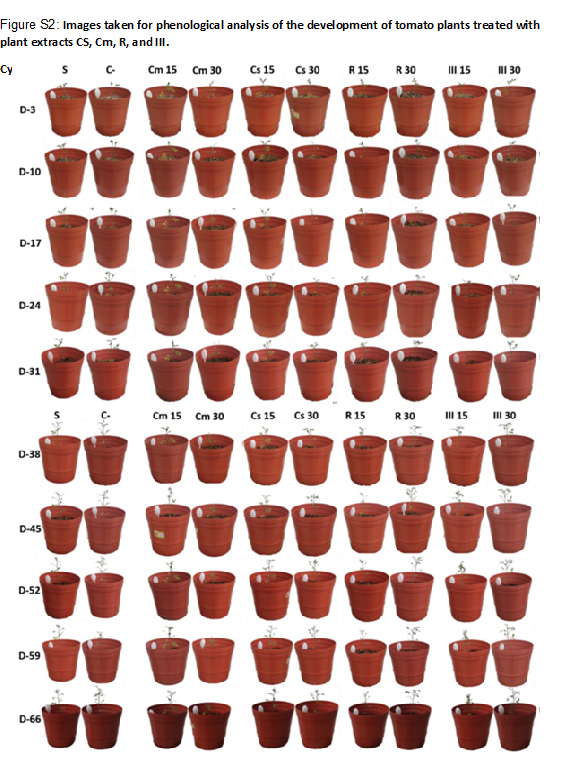

Supplement: Supplementary file 1 [file plants-15-01872-s001.zip › Figure S2.png]

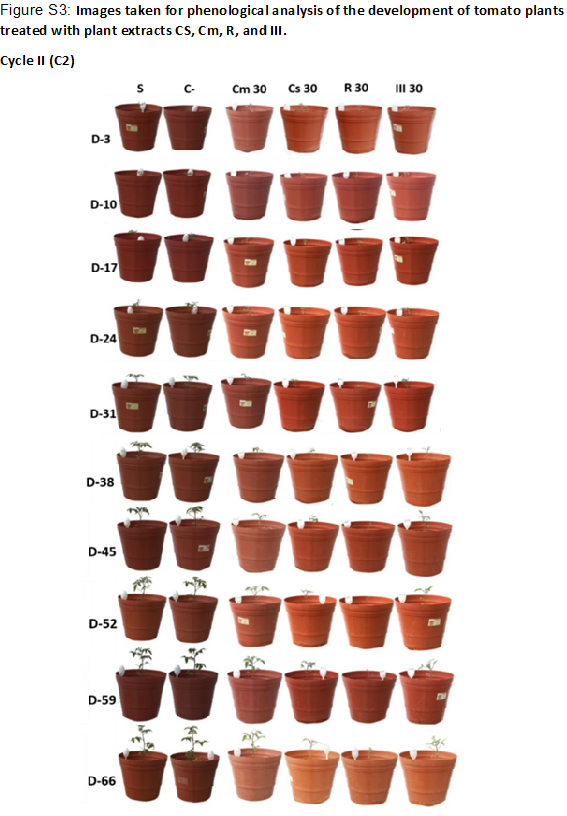

Supplement: Supplementary file 1 [file plants-15-01872-s001.zip › Figure S3.png]
